# Supplementary material for: Exploring the Isomeric Precursors of Olive Oil Major Secoiridoids: An Insight into Olive Leaves and Drupes by Liquid-Chromatography and Fourier-Transform Tandem Mass Spectrometry
Source: Foods. 2021 Aug 31;10(9):2050. doi: 10.3390/foods10092050 (PMC8470314; doi:10.3390/foods10092050)
Supplement: Supplementary file 1 [file foods-10-02050-s001.zip › foods-1348092-supplementary.pdf]

Supplementary Materials

Exploring the isomeric precursors of olive oil major secoiridoids: an insight into olive leaves and drupes by liquid-chromatography and Fourier-transform tandem mass spectrometry

R. Abbattista[1], I. Losito[1,2 *], C.D. Calvano[2,3], T.R.I. Cataldi[1,2]

[1]Dipartimento di Chimica, Università degli Studi di Bari "Aldo Moro", via Orabona 4, 70126 Bari (Italy)

[2]Centro Interdipartimentale SMART, Università degli Studi di Bari "Aldo Moro", via Orabona 4, 70126 Bari (Italy)

[3]Dipartimento di Farmacia e Scienze del Farmaco, Università degli Studi di Bari "Aldo Moro", via Orabona 4, 70126 Bari (Italy)

[*]Corresponding Author, e-mail: ilario.losito@uniba.it, phone: 0039 080 5442506

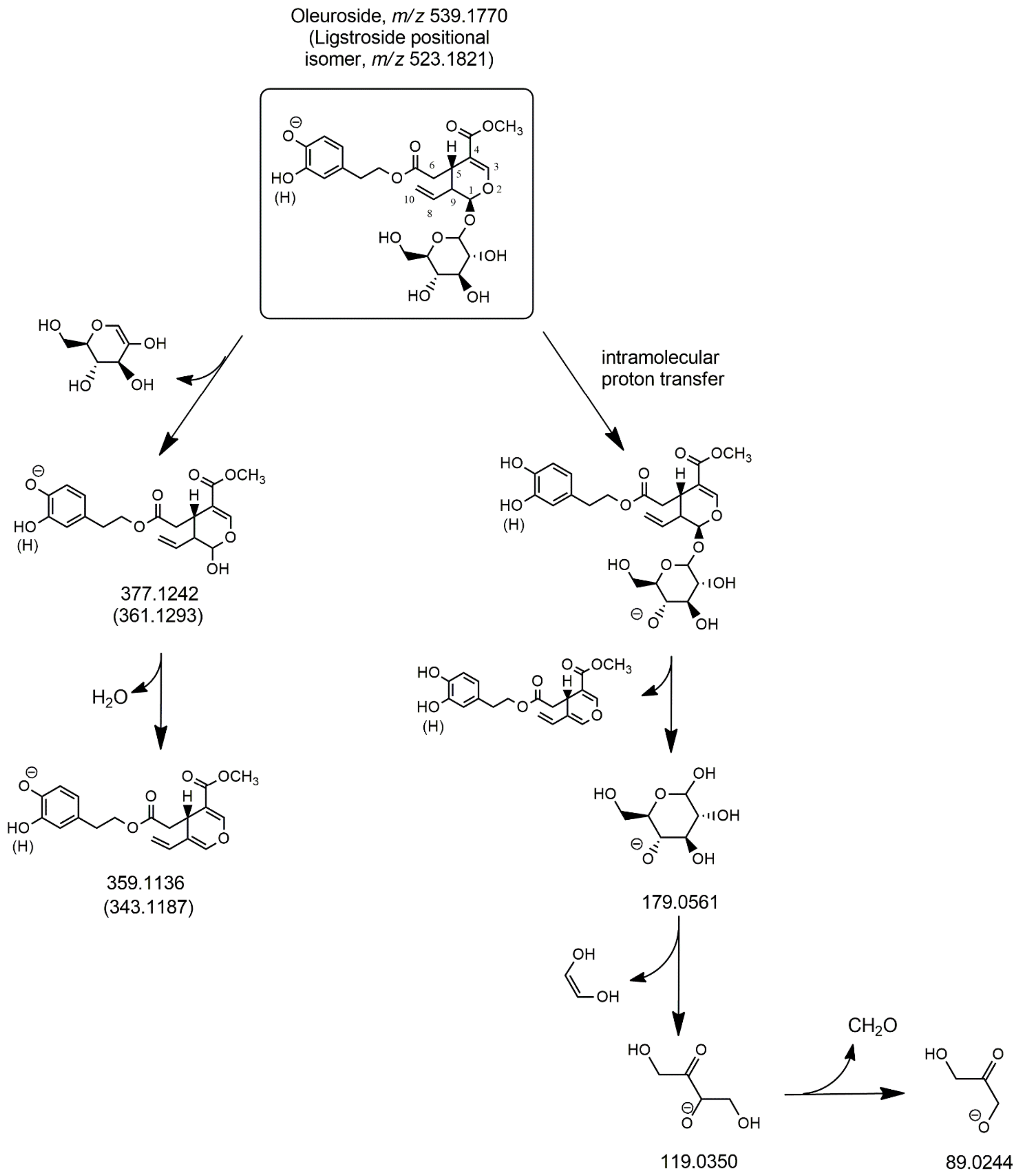

Figure S1. Fragmentation pathways hypothesized for the [M-H]$^-$ ions of oleuroside and of ligstroside positional isomers detected in olive leaves/drupes extracts, all including a C^{10}=C^{8} bond in their structures. *m/z* ratios reported in parentheses are referred to product ions of ligstroside positional isomers, in which a H atom replaces the OH group in the *meta* position of the phenyl ring.

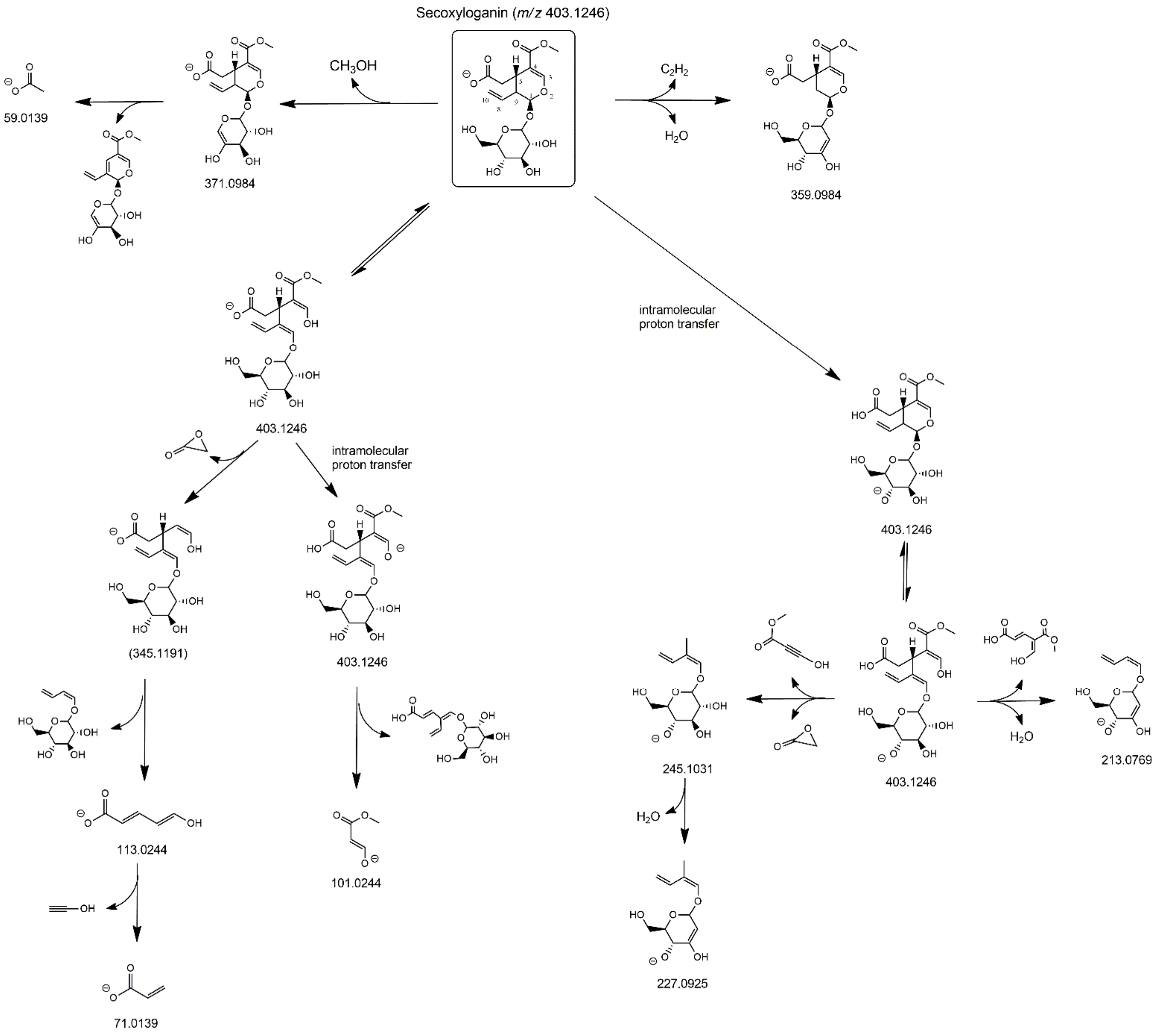

Figure S2. Fragmentation pathways hypothesized for the [M-H]$^-$ ions of secoxyloganin detected in extracts of olive leaves/drupes. Intramolecular proton transfers have been hypothesised to explain the displacement of negative charge from the COOH group to an enolic or a glucose OH group (only one of the possible charging sites is shown in the latter case), required to explain the generation of some product ions.
